# Patients With Symptomatic AAAs Are More Likely to Develop Lumen Partial-Thrombus After Endovascular Aortic Repair Than Asymptomatic Patients

**DOI:** 10.3389/fcvm.2022.848848

**Published:** 2022-03-17

**Authors:** Enci Wang, Xinsheng Xie, Dandan Xu, Xiaolong Shu, Yu fei Zhao, Yuchong Zhang, Peng Lin, Daqiao Guo, Weiguo Fu, Lixin Wang

**Affiliations:** ^1^Department of Vascular Surgery, Xiamen Branch, Zhongshan Hospital, Fudan University, Xiamen, China; ^2^Department of Vascular Surgery, Zhongshan Hospital, Fudan University, Shanghai, China; ^3^Department of Neurology, Quanzhou First Hospital Affifiliated to Fujian Medical University, Quanzhou, China

**Keywords:** abdominal aortic aneurysm, EVAR, symptom, thrombus, predict

## Abstract

**Background:**

According to their symptoms, abdominal aortic aneurysms (AAAs) can be divided into symptomatic and asymptomatic types. This study aimed to explore the differences and correlations between postoperative lumen thrombosis in these two groups after endovascular aortic repair (EVAR).

**Methods:**

A retrospective study using clinical data of 169 patients with AAA treated with EVAR collected in our hospital between January 2018 and January 2021 was conducted based on the inclusion and exclusion criteria for patient selection. Based on whether the patient had clinical symptoms at admission and the presence of a complete lumen thrombus during follow-up, the patients were divided into two sets of groups: a complete-thrombus group (*n* = 44) and a partial-thrombus group (*n* = 125), and a group with clinical symptoms (*n* = 32) and a group without clinical symptoms (*n* = 137). The clinical data of these groups were compared, and a further stratified analysis was performed.

**Results:**

A total of 169 patients were included in the analysis. An abdominal aorta stent graft was successfully implanted in all patients. The complete-thrombus rate of the patients in this study was 73.96%. Univariate analysis showed that the maximal aortic diameter and preoperative peripheral blood neutrophil levels affected the clinical symptoms of patients with AAA (*p* < 0.05). The complete thrombus rate of the lumen of the AAA was lower in patients with clinical symptoms than in those without symptoms during the follow-up period (*p* < 0.05). Female sex, preoperative hyperuricemia, and symptoms at admission were independent risk factors for a partial thrombus in the lumen during follow-up. Based on these independent risk factors, we constructed a scoring system to differentiate patients into low- (0 points), middle- (1 point), and high-risk (2 points) groups. The scoring system could distinguish the complete lumen thrombosis rate after EVAR to a certain extent.

**Conclusions:**

Patients with symptomatic AAAs were more likely to develop incomplete lumen thrombosis than asymptomatic patients during follow-up after EVAR.

## Introduction

An abdominal aortic aneurysm (AAA) is one of the most common aneurysms with an incidence of approximately 5% in the population (1). AAAs have an insidious onset and often have no obvious symptoms before rupture. When the aneurysm ruptures, it becomes life-threatening and a serious disease that threatens human health (2). Since Parodi et al. (3) first reported endovascular aortic repair (EVAR) for the treatment of AAA in 1991, its use has steadily increased. EVAR greatly reduces perioperative mortality and complication rates in AAA patients (4). However, according to one study, re-intervention for residual endoleak or migration was required in about 13–20% of patients after EVAR, and ruptured aneurysms were found in 6% of patients (5). Aneurysmal sac enlargement after EVAR is a significant risk factor for rupture. Several studies have reported the effects of intraluminal thrombus level on aneurysm progression (6, 7). In patients undergoing EVAR, a higher intraluminal thrombus volume decreases the regression of the aneurysmal sac. A complete lumen thrombus can help delay the expansion of the aneurysm sac and it also is one of the important protective factors for delaying the progression of AAA after EVAR (8). Additionally, the effect of intraluminal thrombus levels on the reduction of aneurysm wall stress has been reported in a previous study (9).

The common symptoms of AAA are abdominal and back pain. AAAs can be divided into symptomatic and asymptomatic aneurysms; symptomatic AAAs are relatively rare, accounting for approximately 5–22% (10). Patients with symptomatic AAAs have abdominal and back pain symptoms caused, which may indicate the aneurysm's imminent rupture (11). The results of previous studies generally suggest that the effect of EVAR on symptomatic AAA is inferior to its effect on selective asymptomatic AAA, with an average case mortality of 16% (12). There are also related research findings showing no difference in perioperative results after endovascular repair of symptomatic and asymptomatic AAA (13). This study aimed to investigate the difference between symptomatic and asymptomatic AAAs in the formation of aneurysm cavity thromboses after EVAR.

## Methods

### Participants

**Figure 1:**
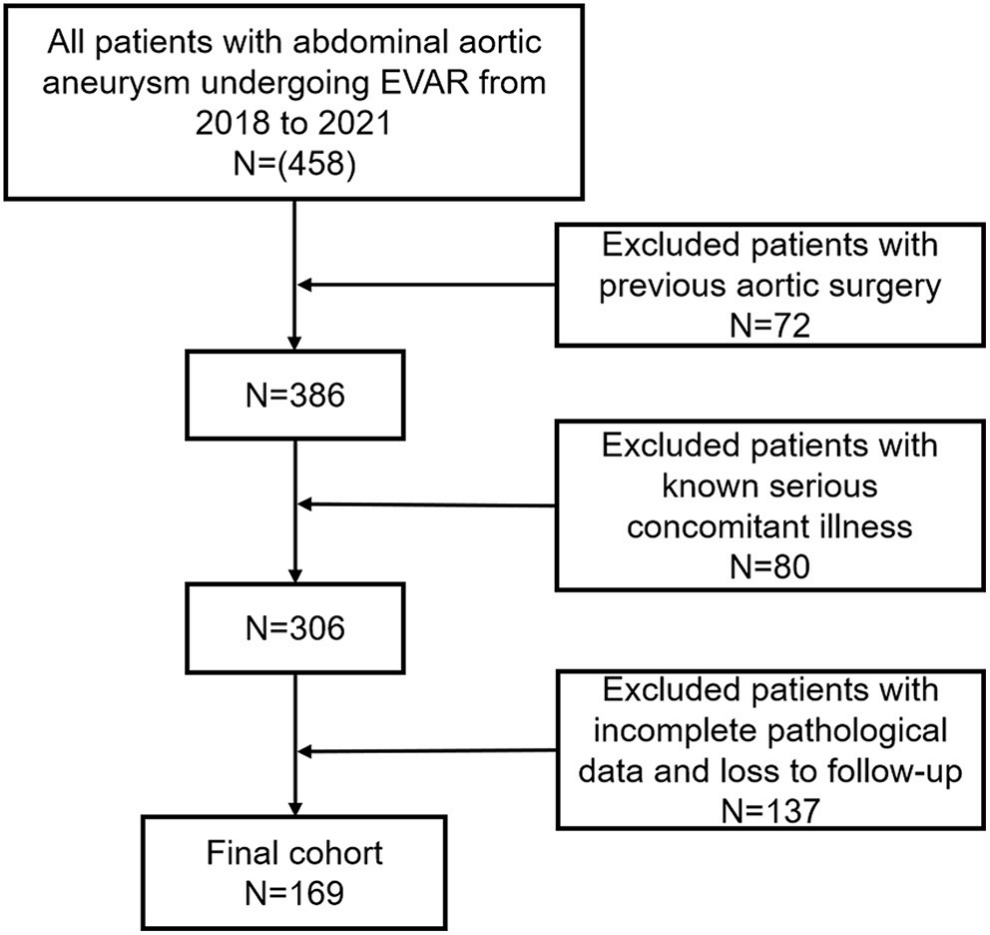
Diagram of study population.

Between January 2018 and January 2021, 169 consecutive patients underwent EVAR for AAAs in our department (Figure 1). All patients were diagnosed preoperatively with AAA using computed tomography angiography (CTA). The inclusion criteria were: patients diagnosed preoperatively with AAA requiring intervention (with anatomical conditions: the length of proximal landing zone for aneurysm sealing is greater than 1.5 cm, the angulation of the aneurysm neck was less than 60°, no thrombosis, no extensive calcification [calcification range <50%]); a maximum lumen diameter of AAA >5.0 cm, or 4.0–5.0 cm with an increase of >0.5 cm in the previous 6 months, or greater than 1.5 times the normal diameter of the aorta inferior to the renal arteries; and an external iliac artery diameter ≥7 mm. The exclusion criteria were: a ruptured AAA; hemodynamic instability; previous aortic surgery; planned major surgery (patients with severe abdominal aortic disease requiring staged treatment or hybrid surgery treatment); incomplete pathological data; death before arrival at the hospital or in the emergency department; and known serious concomitant illness associated with a life expectancy of less than 1 year. Patients with endoleak were also exclude from this study. All patients voluntarily chose EVAR and signed an informed consent form following an explanation of the associated surgical risks. The same group of surgeons performed all the EVAR procedures. The study protocol conformed with the ethical guidelines of the 1975 Declaration of Helsinki.

### Variables and Definitions

**Figure 2:**
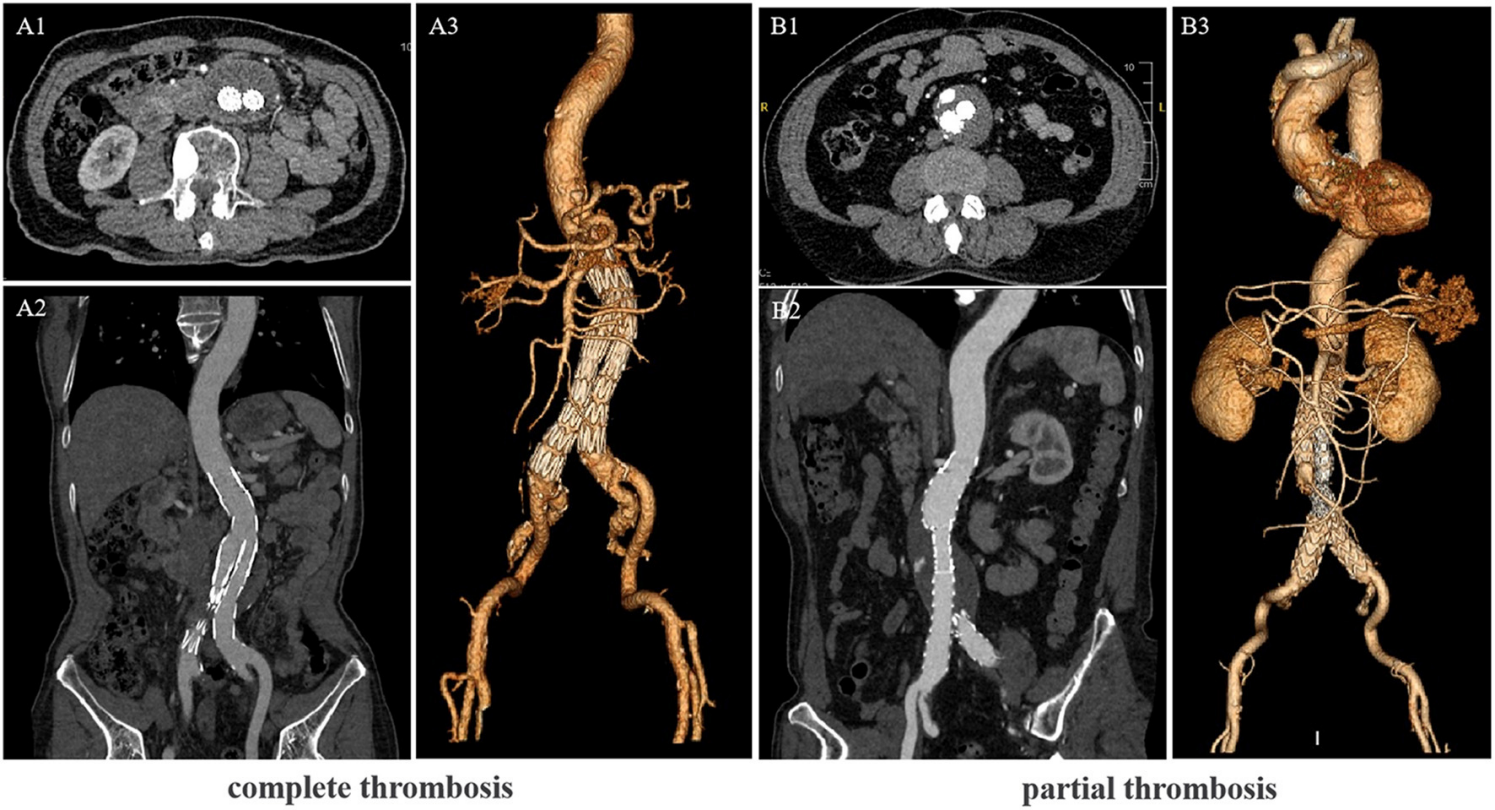
Computed tomography angiography (CTA) scans of the Abdominal aortic aneurysm (AAA) patients after endovascular aortic repair (EVAR). **(A)** CTA showing the lumen complete thrombosis. **(B)** CTA showing the lumen partial thrombosis. **(A1,B1)** Cross-sectional image, **(A2,B2)** vertical-sectional image and **(A3,B3)** CTA 3D reconstruction image.

The study variables included age, gender, acute and concomitant illness at admission (e.g., coronary heart disease, diabetes, hypertension, stroke, and hyperuricemia), the lumen's maximum diameter (from the outer wall to the outer wall), length of hospital stay, and red blood cell, platelet, and neutrophil counts. Hematological specimens were collected within 24 h after admission and within 2 days preoperatively for blood cell count and classification. Symptoms were defined as: patient with back pain or abdominal pain, other possible thoracic and dorsal abdominal diseases were excluded. The level of lumen thrombus was determined by CTA in the follow-up period (Figure 2).

### Follow-Up

Follow-up was conducted by a special person, using an outpatient service, e-mail, and telephone. All patients underwent CTA examination at 1, 3, 6 months and yearly after that to evaluate the lumen thrombosis levels, lumen morphological changes, the presence of endoleak, and the diameter changes in the aneurysm lumen. In this study, the time to calculate the thrombus rate (the time to evaluate the thrombus status) is the time from the day of surgery to the day of CTA at the last follow-up examination in our hospital.

### Statistical Analysis

All statistical analyses were performed using the Statistical Package for Social Science (SPSS) version 23.0 for Windows (IBM, Chicago, IL, USA). The results are expressed as percentages or means ± standard deviation (SD) unless otherwise noted. The distribution of clinicopathological data was summarized using descriptive statistical methods. Data were analyzed using the chi-squared test, Fisher's exact test, or student's *t*-test. Variables were assessed by univariate and multivariate analyses using a logistic regression model. The variables with *P* < 0.05 in the univariate analysis were included in a multivariate binary logistic regression model. The data from the multivariate analysis and the grouping of each risk are shown as odds ratio values with corresponding 95% confidence intervals (CI). The receiver operating characteristic (ROC) curve was calculated to assess the reliability of the scoring system and regression model to predict the complete thrombus after EVAR. The area under the curve (AUC) was also measured, shown as the absolute value and 95% CI. The accumulative complete-thrombus rate was calculated according to the Kaplan-Meier method, and the Kaplan-Meier method was used to draw the complete-thrombus rate curve. The accumulative complete-thrombus rate was compared by the Log-rank test. Differences were considered statistically significant when *P* < 0.05.

## Results

### Patient Population

There were 169 patients with AAA in our cohort, including 142 men (82.1%) and 27 women (17.9%) with an average age of 69.77 ± 7.26 years. Patients with symptomatic AAA accounted for 18.93% of participants in this study. The lumen complete-thrombus rate of the patients in this study was 73.96%. Based on whether the patient had clinical symptoms at admission and the presence of a complete lumen thrombus during follow-up, the patients were divided into two sets of groups: a complete-thrombus group *(n* = 44) and a partial-thrombus (*n* = 125), a group with clinical symptoms (*n* = 32) and a group without clinical symptoms (*n* = 137; Table 1).

**Table 1:** Patient characteristics grouped by the presence or absence of symptom.

	**Overall**		**Symptom**		**No-Symptom**		**p**
	**No**	**%**	**No**	**%**	**No**	**%**	
Overall	169	100	32	100	137	100	-
Age, years, mean (SD)	69.77	7.26	70.88	7.06	69.51	7.30	0.340
**Sex**							0.952
Female	27	16.0	5	15.6	22	16.1	
Male	142	84.0	27	84.4	115	83.9	
**Hypertension**							0.271
Yes	124	73.4	21	65.6	103	75.2	
No	45	26.6	11	34.4	34	24.8	
**Diabetes**							0.195
Yes	29	17.2	3	9.4	26	19.0	
No	140	82.8	29	90.6	111	81.0	
**Coronary heart disease**							0.206
Yes	41	24.3	5	15.6	36	26.3	
No	128	75.7	27	84.4	101	73.7	
**Stroke**							0.282
Yes	13	7.7	1	3.1	12	8.8	
No	156	92.3	31	96.9	125	91.2	
**Hyperuricemia**							0.273
Yes	5	3.0	0	0	5	3.6	
No	164	97.0	32	100	132	96.4	
**Complete-thrombus**							0.011
Yes	125	74.0	18	56.3	107	78.1	
No	44	26.0	14	43.7	30	21.9	
**Type of the endograft**							0928
Endurant	115	68.0	20	62.5	95	69.3	
PERCUTEK	28	16.6	6	18.8	22	16.1	
INCRAFT	11	6.5	3	9.4	8	5.8	
Zenith F	9	5.3	2	6.3	7	5.1	
Excluder	6	3.6	1	3.1	5	3.6	
**Schumacher types**							0.622
I	17	10.1	3	9.4	14	10.2	
IIA	108	63.9	19	59.4	89	65.0	
IIB	32	18.9	6	18.8	26	19.0	
IIC	12	7.1	4	12.5	8	5.8	
Diameter, cm, mean (SD)	47.71	12.13	52.20	10.67	46.70	12.24	0.022
RBC, 10^12^/L, mean (SD)	4.25	0.52	4.33	0.52	4.23	0.52	0.335
Platelet, 10^9^/L, mean (SD)	177.25	52.37	186.25	59.89	175.15	50.46	0.282
Neutrophile, 10^9^/L,mean (SD)	4.13	1.99	5.15	2.65	3.90	1.74	0.015
LOS, days, mean (SD)	2.33	1.49	2.56	2.03	2.27	1.33	0.318
Follow-up, months, mean (SD)	4.21	2.07	4.05	2.09	4.25	2.07	0.622

*RBC, Red blood count; LOS, Length of stay*.

### Univariate and Multivariate Analyses for Symptoms and Complete-Thrombus Rate

The results of the univariate analysis of the symptoms showed that the maximal aortic diameter and preoperative peripheral blood neutrophil levels were factors affecting the clinical symptoms of AAA patients (*P* < 0.05). However, in our study, sex, age, preoperative peripheral blood platelet and red blood cell counts, preoperative hypertension, diabetes, coronary heart disease, hyperuricemia, and stroke history were not significantly correlated with the clinical symptoms (*P* > 0.05). In addition, clinical symptoms did not affect the length of the postoperative hospital stay (*P* > 0.05). Interestingly, we found that symptoms at admission affected the level of postoperative lumen thrombi. The differences in the clinical data between the two patient groups are shown in Table 1.

The results of the univariate analysis showed that sex, preoperative hyperuricemia, and admission with symptoms were significantly related to the thrombus level in the lumen (*P* < 0.05). However, age, preoperative peripheral blood platelet count, neutrophil count, red blood cell count, hypertension, diabetes, coronary heart disease, and stroke history were not significantly correlated with the thrombus level in the lumen (*P* > 0.05). As suggested by the binary logistic analysis, female sex, preoperative hyperuricemia, and symptoms at admission were independent risk factors for a partial thrombus in the lumen. The between-group differences in clinical are shown in Table 2.

**Table 2:** Patient characteristics grouped by the thrombus status after EVAR.

	**Complete-thrombus**		**Partial-thrombus**		**p**	**95% CI**	**p**
	**No**	**%(SD)**	**No**	**%(SD)**			
**Overall**	125		44				
Age, years, mean (SD)	69.60	7.01	70.25	7.97	0.611		
**Sex**					0.017	0.120–0.723	0.008
Female	15	12.0	12	27.3			
Male	110	88.0	32	72.7			
**Hypertension**					0.193		
Yes	95	76.0	29	65.9			
No	30	24.0	15	34.1			
**Diabetes**					0.471		
Yes	23	18.4	6	13.6			
No	102	81.6	38	96.4			
**Coronary heart disease**					0.894		
Yes	30	24.0	11	25.0			
No	95	76.0	33	75.0			
**Stroke**					0.686		
Yes	9	7.2	4	9.1			
No	116	92.8	40	90.9			
**hyperuricemia**					0.005	0.005–0.445	0.008
Yes	1	0.8	4	9.1			
No	124	99.2	40	90.9			
**Symptom**					0.011	0.126–0.682	0.004
Yes	18	14.4	14	11.2			
No	107	85.6	30	88.8			
**Type of the endograft**					0.957		
Endurant	84	67.2	31	70.5			
PERCUTEK	21	16.8	7	16.0			
INCRAFT	9	7.2	2	4.5			
Zenith F	7	5.6	2	4.5			
Excluder	4	3.2	2	4.5			
**Schumacher types**					0.568		
I	12	9.6	5	11.4			
IIA	82	65.6	26	59.1			
IIB	21	16.8	11	25.0			
IIC	10	8.0	2	4.5			
Diameter, cm, mean (SD)	47.26	11.93	49.03	12.72	0.412		
RBC, 10^12^/L, mean (SD)	4.29	0.51	4.13	0.52	0.090		
Platelet, 10^9^/L, mean (SD)	179.76	52.57	170.14	51.72	0.296		
Neutrophile, 10^9^/L,mean (SD)	4.09	1.96	4.25	2.10	0.646		
Follow-up, months, mean (SD)	4.22	2.00	4.18	2.29	0.917		

*RBC, Red blood count; LOS, Length of stay*.

### Risk Gradation of Partial-Thrombus and Construction of the Predictive Scoring System

Accumulative complete thrombus rate analysis was performed on all patients to clarify whether clinical symptoms during the follow-up period affected the thrombus level of the lumen of abdominal AAA. The complete thrombus rate of the AAA's lumen was lower in patients with clinical symptoms than in those without symptoms during the follow-up period (*p* < 0.05; Figure 1). The risk factors were simplified to the following weights based on their regression coefficients: female, 1 point; hyperuricemia, 1 point; and clinical symptoms, 1 point. Based on these scores, the patients were divided into low- (0 points), middle- (1 point), and high-risk (2 points) groups. Of the total patients, the low-risk groups comprised 65.09%; the middle-risk group, 31.95%; and the high-risk group, 2.96%. The non-complete thrombus rates were 15.45, 44.44, and 60.0% in the low-, middle-, and high-risk groups, respectively. Compared to the low-risk patients (reference group), the odds ratio for incomplete thrombus was 4.39 (95% CI: 0.108–0.481, *P* < 0.01) and 8.20 (95% CI: 0.019–0.785, *P* = 0.01) in the middle- and high-risk patients, respectively (Table 3).

**Table 3:** Risk gradation of the incomplete thrombus.

**Risk gradation**	**Risk factor score**	**Case number (*n*, %)**	**No-complete thrombus rate (*n*, %)**	**OR**	**95% CI**	***P***
Low-risk	0	110 (65.09)	17 (15.45)	1	-	-
Middle-risk	1	54 (31.95)	24 (44.44)	4.39	0.108–0.481	0.000
High-risk	2	5 (2.96)	3 (60.0)	8.20	0.019–0.785	0.010

### Effectiveness Test of the Predictive Scoring System

**Figure 3:**
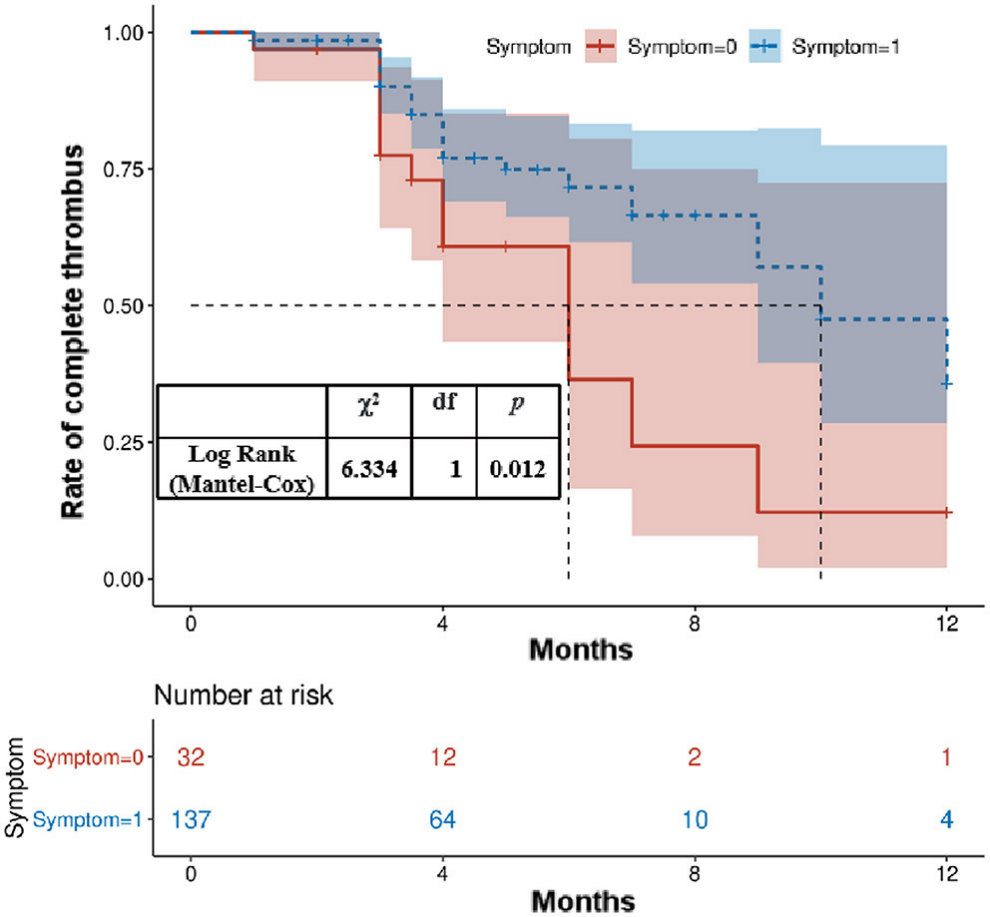
Kaplan–Meier analysis for the complete thrombosis rate according to the presence of clinical symptoms at admission.

**Figure 4:**
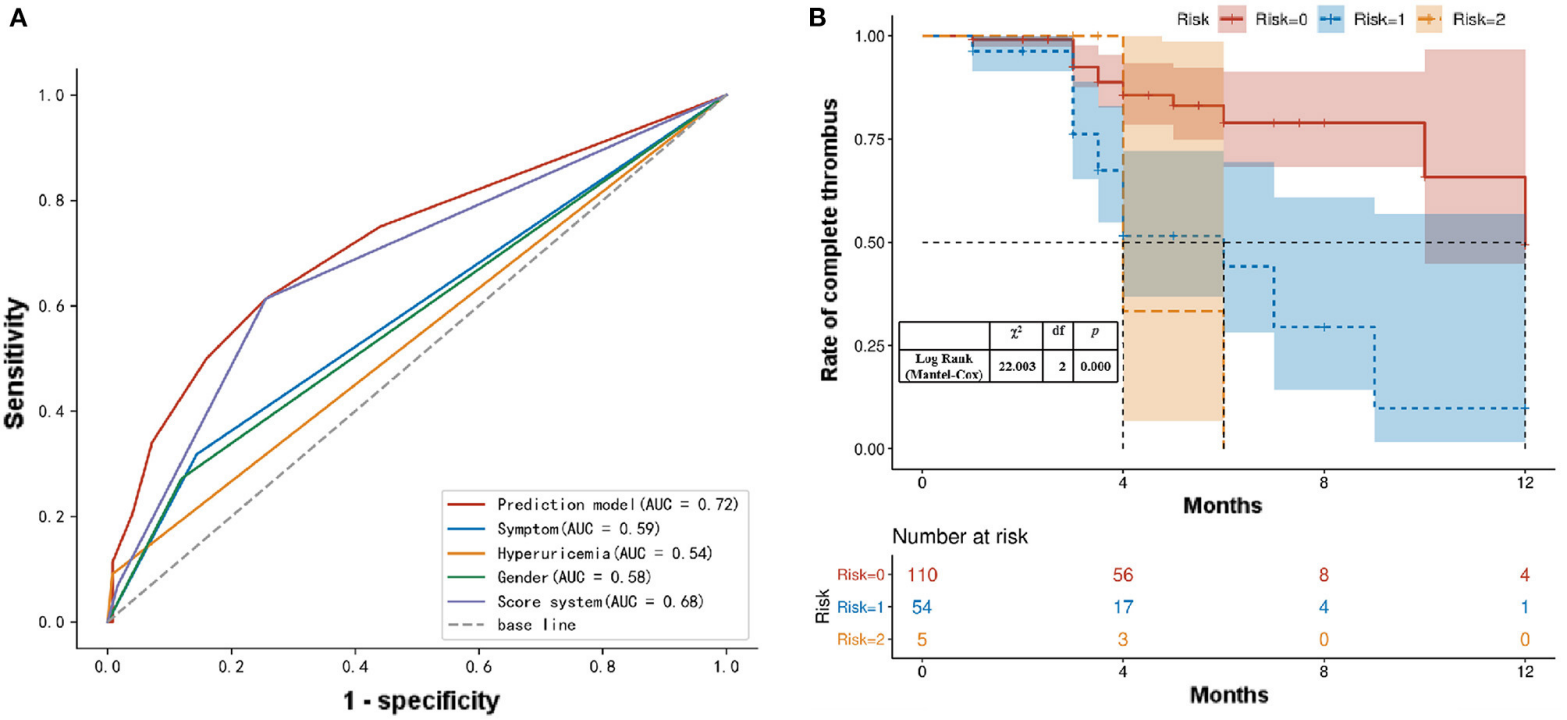
**(A)** ROC analysis of the sensitivity and specificity of the predictive value of a scoring system for AAA patients with EVAR. ROC, Receiver operating characteristic; AAA, abdominal aortic aneurysms; EVAR, endovascular aortic repair. **(B)** Kaplan–Meier analysis for the complete thrombosis rate according to the risk gradation.

To further investigate whether the predictive efficacy of our system increased when the three factors (female sex, hyperuricemia, and admission with symptoms) were combined, we analyzed the predictive value of the combined independent risk factor (in the logistic regression model) to determine the non-complete thrombus rate using an ROC curve analysis (Figure 3). The area under the ROC curve indicated that female sex and hyperuricemia combined with symptoms at admission resulted in a more accurate predictive value for the non-complete thrombus rate in AAA patients after EVAR. The area under the curve of our scoring system was 0.683 (95% CI: 0.587–0.778; Figure 4A). In addition, we performed an accumulative complete thrombus rate analysis according to risk gradation. The results showed that the risk gradation based on the scoring system we constructed could better distinguish the level of incomplete thrombosis in the lumen during follow-up (Figure 4B).

## Discussion

With the globally aging population, the incidence of AAA is increasing, and endovascular repair has become the main treatment for AAA (14). Although EVAR can significantly reduce the perioperative mortality and complication rates in patients with AAA, long-term follow-up data showed that EVAR resulted in a higher re-intervention rate than traditional open surgery due to its unique stent-related complications (including endoleak, stent displacement) (15). Abdominal aneurysmal sac enlargement after EVAR is a critical issue. AAA sac enlargement after EVAR was 41% at 5 years, which increased over the study period (16). Complete thrombosis of the aneurysm sac after EVAR for an AAA can form a stable white thrombus, which isolates the aneurysm lumen and reduces the tension and pressure of the aneurysm wall, thereby preventing the formation of endoleaks and continuous expansion of the aneurysm.

In this study, patients with symptomatic AAA accounted for 18.93% of participants, similar to the 5–23% reported in the literature (16). Our study found that the complete thrombus rate in the lumen of the AAAs was lower in patients with clinical symptoms than in those without symptoms. In addition, compared with asymptomatic patients, patients with symptomatic AAAs tended to have larger aortic diameters and higher neutrophil counts. Previous studies have shown that aneurysm diameter is the strongest predictor of rupture. The rupture of asymptomatic AAAs with a diameter <5 cm is rare, and the risk significantly increases when the diameter is >5.5 cm (11). Larger aneurysm diameter and faster aneurysm expansion are related to AAA symptoms. Studies have shown that inflammation plays a vital role in the pathogenesis of AAA. Inflammatory cells mediate the degradation of extracellular matrix and vascular smooth muscle cells by stimulating proteases, leading to the destruction of the aortic wall (17). During AAA's progression, the corresponding symptoms occur as the AAA cavity's diameter increases. Pain often causes the body to produce a stress response, increasing the white blood cell and corresponding neutrophil counts, consistent with our results (1).

In addition, our research results showed that female sex, preoperative hyperuricemia, and symptoms at admission were independent risk factors for partial thrombus in the lumen. EVAR isolates the tumor sac from the blood circulation, which reduces the pressure in the aneurysm sac, promotes thrombosis and mechanical contraction in the tumor sac, reduces the expansion of the aorta, stabilizes the remodeling of the tumor sac, and reduces the risk of aneurysm rupture. Patients with acute aortic dissection with post-EVAR lumen remodeling were significantly better than those with chronic aortic dissection (18). This may be attributed to the difference in formation time between the two groups; the false cavities of the chronic group formed over a prolonged period, and the false cavity's inner wall became fibrotic, thus making thrombus formation difficult. However, the false cavity of the acute group had a shorter formation time, and the inner wall was rough and easily formed a thrombus (16). Similarly, patients with symptomatic AAAs tended to have a longer illness than patients with asymptomatic AAAs. The tumor cavity is subjected to blood flow for a long time, and fibrosis of the aorta's inner wall makes it difficult to form thrombi.

In addition, under normal circumstances, patients with symptomatic AAAs have larger tumor cavity diameters and volumes than those with asymptomatic AAAs, and it may take longer to achieve complete thrombosis. Lopera et al. also found that the diameter of the aorta correlated with the degree of thrombosis of the false lumen (19). A study on intracranial aneurysms showed that most patients with fully thrombotic intracranial aneurysms were women, with a male-to-female ratio of 1:2 (20). In addition, studies have confirmed that women with AAA are at a higher risk of rupture than men, and smokers and untreated hypertensive patients also have an increased risk of rupture. The increased risk of rupture in women may be attributed to a relatively large aortic size index (21, 22). We speculate that the level of thrombosis in female patients after EVAR may be similarly affected. Hyperuricemia is often accompanied by damage to microvessels and large blood vessels and is an important risk factor for hypertension, coronary heart disease, and metabolic diseases (23). An increasing number of studies have suggested that hyperuricemia is associated with AD and AA (24). Patetsios et al. (25) proposed that uric acid may be associated with aortic dilatation and coronary heart disease. Elevated serum uric acid concentrations were observed in individuals with aortic dilatation, and as the concentration of uric acid increased, the rate of cell apoptosis also increased significantly. Hyperuricemia can damage vascular smooth muscle cells and endothelial cells and promote the occurrence and development of AA and AD (26). Esen et al. (27) found that serum UA concentration was significantly positively correlated with aortic dilation. The level of hyperuric acid in the blood of patients with AAA after EVAR is not corrected in time; therefore, it is speculated that the damaging effect of hyperuric acid on the arteries still exists, thereby affecting the process of tumor cavity thrombosis.

EVAR has become a fundamental option in the treatment of AAA, and several different types of endografts have been proposed to seal aneurysmal sac over time, which allowed symptomatic, asymptomatic, as well as fragile patients be treated in daily clinical practice, thanks to the use of novel endograft with specifical technical characteristics and lower profiles (28–30). EVAR remains an appropriate therapeutic approach based on the comprehensive pre-operative evaluation and post-operative surveillance even in the management of the elderly patients (31, 32). All patients were non-complex abdominal aortic aneurysms in our study, and had similar treatment strategy and stent-graft implantation. And the score system we established can still effectively identify partial thrombosis after EVAR. These findings will help clinicians closely observe and identify AAA patients prone to incomplete thrombus formation during the follow-up, and thus help to develop individualized interventions for better result.

## Limitations

This study has some limitations. First, we excluded patients with other inflammatory diseases (such as Behcet's disease) to ensure consistency before blood collection. Therefore, the results of this study may not be suitable for the clinical treatment of patients with AAA who also suffer from inflammatory diseases. Second, due to the small number of patients with acute rupture, in this study, rupture or the influence of emergency surgery on lumen thrombus levels after EVAR could not be performed. Third, the sample size of this study was small; the limitation of inclusion and exclusion criteria, and the lack of detailed clinical data collection, led to an incomplete analysis of risk factors and failed to find other potential risk factors affecting lumen thrombus levels after EVAR. Fourth, the use of anticoagulant drugs in AAA patients was not clearly registered, some patients did not take medication continuously; some patients took Rivaroxaban orally, measuring 10–20 mg; INR of some patients with oral Warfarin did not fully meet the standard. So we did not include these data in order to avoid confusing the analysis results of our study due to the inclusion of such unclear data and the analysis of the relating data could not be carried out in this study. Fifth, it is a pity that we didn't have enough sample size, there are no patients with these three risk factors at the same time, so the constructed scoring system lacks 3-point patients. To a certain extent, our score system can still effectively identify partial thrombosis after EVAR during follow-up. For the high-risk population of partial thrombosis after EVAR, it is believed that with the further expansion of the research population, the score system can play a more accurate and clinically applicable value.

## Conclusion

Patients with symptomatic AAAs are more likely to develop incomplete lumen thromboses after EVAR than their asymptomatic counterparts during follow-up. Furthermore, the findings of this study could help clinicians identify AAA patients prone to incomplete thrombus formation and help guide individualized treatment of patients with AAA.

## Data Availability Statement

The raw data supporting the conclusions of this article will be made available by the authors, without undue reservation.

## Ethics Statement

The studies involving human participants were reviewed and approved by Ethics Committee of Zhongshan Hospital affiliated to Fudan University. The patients/participants provided their written informed consent to participate in this study.

## Author Contributions

EW performed the literature research and wrote a part of the manuscript. XX contributed to the ideas of the manuscript and wrote the first draft. DX wrote a part of the manuscript. YZ and XS designed the tables. YZ and PL draw the figures. DG provided critical feedback. WF and LW guide the writing. All the authors reviewed the manuscript and approved the submitted version.

## Funding

This work was supported by the National Natural Science Foundation of China (grant number: 81970412), Shanghai Municipal Science and Technology Commission Innovation Fund (grant number: 18441902400), Xiamen Municipal Health Science and Technology Program Fund (grant number: 3502Z20194034), Zhongshan Hospital's Talents Supporting Plan (grant number: 2019ZSGG11), and Xiamen Medical and Health Guidance Project (grant number: 3502220214201088).

## Conflict of Interest

The authors declare that the research was conducted in the absence of any commercial or financial relationships that could be construed as a potential conflict of interest.

## Publisher's Note

All claims expressed in this article are solely those of the authors and do not necessarily represent those of their affiliated organizations, or those of the publisher, the editors and the reviewers. Any product that may be evaluated in this article, or claim that may be made by its manufacturer, is not guaranteed or endorsed by the publisher.
